# Stereotactic radiosurgery for brain metastases from human epidermal receptor 2 positive breast Cancer: an international, multi-center study

**DOI:** 10.1007/s11060-024-04775-3

**Published:** 2024-08-27

**Authors:** Stylianos Pikis, Georgios Mantziaris, Maria Protopapa, Salem M. Tos, Roman O. Kowalchuk, Richard Blake Ross, Chad G. Rusthoven, Manjul Tripathi, Anne-Marie Langlois, David Mathieu, Cheng-Chia Lee, Huai-che Yang, Selcuk Peker, Yavuz Samanci, Michael Yu Zhang, Steve E. Braunstein, Zhishuo Wei, Ajay Niranjan, Dade L. Lunsford, Jason Sheehan

**Affiliations:** 1 Department of Radiotherapy and Stereotactic Radiosurgery, Mediterraneo Hospital, Athens, Greece; 2 Department of Neurological Surgery, University of Virginia Health System, University of Virginia, 1215 Lee St, Charlottesville, VA 22908 USA; 3 Department of Radiation Oncology, Mayo Clinic, Rochester, MN USA; 4 Department of Radiation Oncology, University of Colorado, Aurora, CO USA; 5 Department of Neurosurgery and Radiotherapy, Postgraduate Institute of Medical Education and Research, Chandigarh, India; 6 Department of Neurosurgery, Centre de recherche du CHUS, Université de Sherbrooke, Sherbrooke, QC Canada; 7 Department of Neurosurgery School of Medicine, Neurological Institute, Taipei Veteran General Hospital, Taipei, Taiwan; 8 Department of Neurosurgery, Koc University School of Medicine, Istanbul, Turkey; 9 Department of Radiation Oncology, University of California, San Francisco, CA USA; 10 Department of Neurological Surgery, University of Pittsburgh, Pittsburgh, PA USA

**Keywords:** Radiosurgery, Brain metastasis, Breast cancer, Gamma Knife

## Abstract

**Purpose:**

To report patient outcomes and local tumor control rates in a cohort of patients with biopsy-proven HER-2 positive breast cancer treated with stereotactic radiosurgery (SRS) for brain metastases (BM).

**Methods:**

This international, retrospective, multicenter study, included 195 female patients with 1706 SRS-treated BM. Radiologic and clinical outcomes after SRS were determined and prognostic factors identified.

**Results:**

At SRS, median patient age was 55 years [interquartile range (IQR) 47.6–62.0], and 156 (80%) patients had KPS ≥ 80. The median tumor volume was 0.1 cm^3^ (IQR 0.1–0.5) and the median prescription dose was 16 Gy (IQR 16–18). Local tumor control (LTC) rate was 98%, 94%, 93%, 90%, and 88% at six-, 12-, 24-, 36- and 60-months post-SRS, respectively. On multivariate analysis, tumor volume (*p* = < 0.001) and concurrent pertuzumab (*p* = 0.02) improved LTC. Overall survival (OS) rates at six-, 12-, 24-, 36-, 48-, and 60-months were 90%, 69%, 46%, 27%, 22%, and 18%, respectively. Concurrent pertuzumab improved OS (*p* = 0.032). In this patient subgroup, GPA scores ≥ 2.5 (*p* = 0.038 and *p* = 0.003) and rare primary tumor histologies (*p* = 0.01) were associated with increased and decreased OS, respectively. Asymptomatic adverse radiation events (ARE) occurred in 27 (14.0%) and symptomatic ARE in five (2.6%) patients. Invasive lobular carcinoma primary (*p* = 0.042) and concurrent pertuzumab (*p* < 0.001) conferred an increased risk for overall but not for symptomatic ARE.

**Conclusion:**

SRS affords effective LTC for selected patients with BM from HER-2 positive breast cancer. Concurrent pertuzumab improved LTC and OS but at the same time increased the risk for overall, but not symptomatic, ARE.

**Supplementary Information:**

The online version contains supplementary material available at 10.1007/s11060-024-04775-3.

## Introduction

Human epidermal growth factor receptor 2 (HER-2) is overexpressed in approximately 15-20% of patients with breast cancer, and HER-2 positivity is a risk factor for brain metastases. Brain metastases occur in almost 2% of patients with early HER-2 positive breast cancer (BC) at first recurrence and in up to 50% of patients with metastatic HER2-positive BC patients during the course of their illness [1–5]. The higher incidence of brain metastases may be explained by the improved overall survival afforded with the development of HER-2 targeted therapies and by the biologic propensity of HER-2 positive breast cancer to metastasize to the brain [1, 2, 6].

Current management of brain metastases from HER-2 positive breast cancer includes surgical resection with adjuvant radiotherapy, and radiotherapy alone or stereotactic radiosurgery (SRS). SRS alone has been established as a safe and effective local treatment of brain metastases. It is also used for adjuvant management after prior surgery or failed fractionated radiation therapy [7–9]. In this multi-center retrospective study, we evaluated the safety and efficacy of SRS for brain metastases from HER2-positive primary breast cancer during an 18-year interval.

## Methods

### Study design and population

Eight centers from five countries contributed clinical and imaging data for 195 females who underwent SRS for 1706 intracranial metastases from HER-2 positive breast cancer primary in the period between 2003 and 2021. Each participating center obtained approval to participate in the study by the local institutional review board. Due to the retrospective nature of the study, patient informed consent was waived.

Patient inclusion criteria were (1) tissue diagnosis of HER-2 positive breast carcinoma, (2) presence of one or more brain metastases on MRI, (3) treatment of brain metastasis with SRS, and (4) at least one clinical and neuro-imaging follow-up after SRS.

Data collected included patient demographics, breast cancer histologic diagnosis, disease burden, SRS-treatment parameters, additional treatments after SRS, and follow-up clinical and radiologic outcomes. The graded prognostic assessment (GPA) was determined at the time of intracranial disease diagnosis. SRS concurrent with systemic therapy was defined as: (1) SRS while the patient was actively receiving systemic therapy or (2) SRS delivered within 4-weeks from systemic therapy initiation [10].

### Radiosurgery procedure

SRS was delivered using the Gamma Knife (Elekta AB, Stockholm, Sweden) technology available at each center at the time of treatment. In this study, radiosurgery was delivered as single-session frame-based approach in most of the patients. Using local anesthesia and/or monitored sedation, all patients underwent placement of a stereotactic frame (Leksell frame; Elekta Instruments AB, Stockholm, Sweden). Following frame placement, stereotactic thin sliced (1 mm thick) axial, and coronal pre- and post-contrast enhanced brain magnetic resonance imaging (MRI) or contrast enhanced brain CT, when MRI was contraindicated was obtained for treatment planning.

At centers equipped with the Gamma Knife Icon, Mask-based, hypo-fractionated (i.e. 2 to 5 session) stereotactic radiosurgery was used for some larger volume tumors or those located adjacent to critical neurovascular structures. Prior to the procedure, a stereotactic brain MRI was obtained and an individually molded thermoplastic mask was manufactured. A localization cone beam CT scan was obtained prior to each procedure. During treatment, patient motion was monitored via a fiducial marker placed on the patient’s nose through a nasal outlet on the mask.

The treatment plan (prescription dose, prescription isodose line, and fractionation) was decided by the local radiosurgery team usually comprised by a neurosurgeon, a radiation oncologist, and a medical physicist [9].

### Radiological follow-up and radiographic assessment

Intracranial response and radiation-induced toxicity were evaluated using brain MRI or CT when MRI was contraindicated. Clinical follow-up typically occurred at the time of radiological assessment. The follow-up imaging intervals were performed according to each institution’s protocol, typically every 3 months, or earlier in case of neurological decline. Time to local progression was defined as the period between the time point of smallest lesion volume to the point when > 20% increase in lesion volume was noted on image. Time of intracranial progression was defined as the time point on which a new intracranial lesion or local progression occurred. Leptomeningeal disease was evaluated and diagnosed with cytological cerebrospinal fluid studies or leptomeningeal enhancement on brain MRI according to institutional policies. Radiation toxicity was scored using the Radiation Therapy Oncology Group (RTOG) scale [11].

### Outcomes

The primary endpoint of this study was local tumor control, defined as tumor stability or volumetric regression by over 20% at last imaging follow-up [9]. Secondary endpoints were progression-free and post-SRS survival.

### Statistical analysis

All statistical analyses were performed using R programming in R Studio [12, 13]. All analyses are presented in a per patient or per lesion basis, according to context. Univariate and multivariate analyses were performed for outcome measures using Cox regression analysis. Time-dependent analyses for local control, progression-free and overall survival were performed using Kaplan-Meier. Metastases were censored for local response at last brain imaging or at the time of additional treatment of the metastasis for reasons other than local SRS failure. The differences between the function curves were analyzed using the log-rank test. *P* < 0.05 was defined as statistically significant and missing data were not imputed.

### Cohort and tumor characteristics

A total of 195 female patients with 1706 SRS-treated intracranial metastases from HER-2 positive primary breast cancer were included in the study. The primary histologic diagnosis was invasive ductal carcinoma in 152 (82.6%) patients, other in 23 (12.5%) and, invasive lobular carcinoma in 9 (4.9%) patients. Primary tumors were estrogen receptor (ER) positive and progesterone-receptor (PR) positive in 50.3% and 35.1% of the cases respectively (Table 1). At SRS, the median patient age was 55 years [ (IQR) 47.6–62.0] and extracranial disease was present in 149 (76.4%) patients. At the time of radiosurgery, 156 patients had KPS ≥ 80. The median time from primary to brain metastasis diagnosis was 35 months (IQR 15.0–65.0) and in 17 (8.7%) patients, intracranial disease was present at diagnosis of the primary breast cancer. The median brain metastases volume was 0.1 cm^3^ (IQR 0.1–0.5) (Table 1).

**Table 1:** Patient clinical outcomes and tumor characteristics

	*N* = 195^*1*^
Histology	
Invasive ductal carcinoma	152 (82.6%)
Invasive lobular carcinoma	9 (4.9%)
Other	23 (12.5%)
Not available	11 (5.6%)
Estrogen receptor positive	97 (50.3%)
Not available	2 (1%)
Progesterone receptor positive	67 (35.1%)
Not available	4 (2.05%)
Systemic disease status	
Extracranial metastases	149 (76.4%)
No extracranial metastases	46 (23.6%)
Stage at diagnosis	
I	25 (13.0%)
II	26 (13.5%)
III	54 (28.1%)
IV	87 (45.3%)
Not available	3 (1.5%)
Brain metastases at diagnosis	17 (8.7%)
KPS at SRS	
60	4 (2.1%)
70	32 (16.7%)
80	71 (37.0%)
90	64 (33.3%)
100	21 (10.9%)
Not available	3 (1.5%)
Age at SRS	55.0 (47.4,62.0)
Intracranial progression	117 (60.0%)
Leptomeningeal dissemination	32 (16.4%)
Time to LMD, months	12.2 (5.1,22.1)
Adverse radiation events	
Asymptomatic ARE	27 (13.8%)
Asymptomatic hemorrhage	1 (0.5%)
No	162 (83.1%)
Symptomatic ARE	5 (2.6%)
Time to ARE, months	6.0 (3.0,30.0)
New CN palsy	
No	144 (98.6%)
Permanent	1 (0.7%)
Transient	1 (0.7%)
Not available	49 (25.1%)
New neurological deficit	18 (11.9%)
Not available	44 (22.5%)
Additional radiation	88 (45.8%)
Not available	3 (1.5%)
Time to additional radiation	9.4 (4.5,12.9)
Additional surgery	24 (12.4%)
Not available	2 (1%)
Time to additional surgery	15.9 (5.8,21.8)
Status at last follow-up	
Alive	64 (32.8%)
Dead	131 (67.2%)
Clinical follow-up, (months)	17.0 (9.0,31.5)
Radiographic follow-up, (months)	14.5 (6.0,27.4)
Progression-free survival, (months)	8.9 (5.0,15.8)
Overall survival from primary diagnosis, (months)	66.3 (40.5,103.8)
Overall survival from BM diagnosis, (months)	22.0 (12.5,38.3)
Time to brain metastases diagnosis, (months)	35.0 (15.0,65.0)
Number of treated metastases	3.0 (1.0,7.5)

^*1*^n (%); Median (25%,75%)

### Systemic treatments

Prior to SRS, 119 (61%) of the patients received systemic chemotherapy; targeted therapy with pertuzumab, trastuzumab, lapatinib, and emtasine-trastuzumab was administered to 44 (22.6%), 100 (51.3%), 31 (15.9%), and 2 (1.0%) patients, respectively. Concurrent with SRS, 53 (27.2%) patients were treated with trastuzumab, 22 (11.3%) with pertuzumab, 18 (9.2%) with lapatinib, and 6 (3.1%) with emtasine-trastuzumab. At SRS, 34 (17.4%) patients received systemic chemotherapy (Table 2).

**Table 2:** Systemic and local therapies administered

Pertuzumab administration	
After SRS	24 (12.4%)
Before SRS	37 (19.1%)
Concurrent	22 (11.3%)
Not administered	111 (57.2%)
Not available	1 (0.5%)
Lapatinib administration	
After SRS	35 (18.0%)
Before SRS	28 (14.4%)
Concurrent	18 (9.3%)
Not administered	113 (58.2%)
Not available	1 (0.5%)
Trastuzumab administration	
After SRS	53 (27.2%)
Before SRS	60 (30.8%)
Concurrent	53 (27.2%)
Not administered	29 (14.9%)
Emtasine trastuzumab administration	
After SRS	18 (9.3%)
Before SRS	2 (1.0%)
Concurrent	6 (3.1%)
Not administered	168 (86.6%)
Not available	1 (0.5%)
Immunotherapy administration	
After SRS	2 (1.1%)
Not administered	179 (98.9%)
Not available	14 (7.1%)
Chemotherapy administration	
After SRS	67 (34.4%)
Before SRS	68 (34.9%)
Concurrent	34 (17.4%)
Not administered	26 (13.3%)
Additional radiation	88 (45.8%)
Time to additional radiation	9.4 (4.5,12.9)
Additional surgery	24 (12.4%)
Time to additional surgery	15.9 (5.8,21.8)

### SRS parameters

Single-session SRS was utilized for 1,695 (99.4%) brain metastases and hypo-fractionated SRS for 11 lesions. Of these, six (0.4%) lesions were managed with five-fraction SRS and five (0.3%) were managed with three-fraction SRS. The median prescription dose was 16 Gy (IQR 16–18) and the median maximum dose was 22.1 Gy (IQR 19.0-28.6) (suppl. Table 1).

## Results

### Local control

**Fig. 1:**
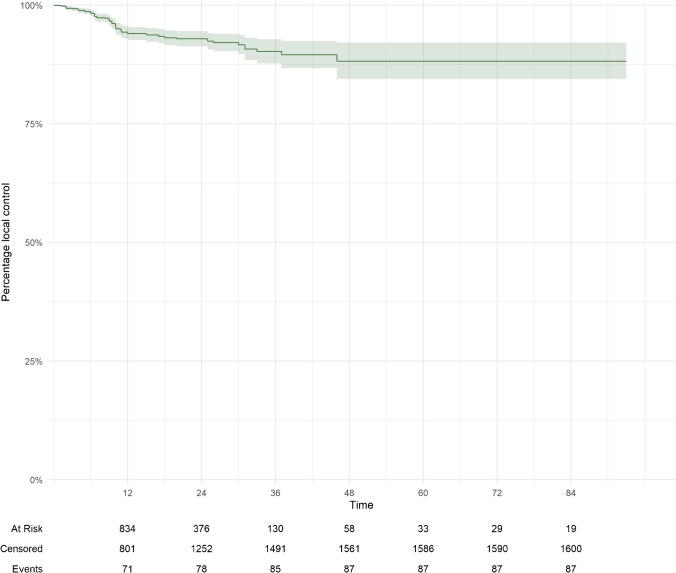
Local tumor control via the Kaplan–Meier curve

The median radiological follow up was 14.5 (IQR 6.0-27.4) months and follow-up was available for all patients. Local tumor control, defined as stability or regression, was 98% (95%CI = 98–99%), 94% (95%CI = 93–95%), 93% (95%CI = 91-95%), 90% (95%CI = 88-93%), and 88% (95%CI = 84-92%) at six-, 12-, 24-, 36- and 60-months post-SRS, respectively (Fig. 1). At the last imaging follow up, local progression of 87 (5.1%) SRS-treated lesions was noted. On multivariate analysis, tumor volume (HR = 1.1; 95%CI = 1.07–1.14; *p* = < 0.001) and pertuzumab administration concurrent with SRS were associated with improved local control (HR = 0.42; 95%CI = 0.19–0.92; *p* = 0.02) (suppl. Table 2). The intracranial progression-free survival (PFS) was 8.9 months (IQR 5.0–15).

### Clinical outcomes and post-SRS overall survival

The median clinical follow-up was 17.0 months (IQR 9.0-31.5) and data were available for all patients. New neurologic deficits after SRS were reported in 18 (11.9%) patients. One patient (0.7%) developed permanent, and one patient (0.7%) developed transient cranial nerve palsy. There was one case (0.5%) of asymptomatic post-SRS intra-cranial hemorrhage.

**Fig. 2:**
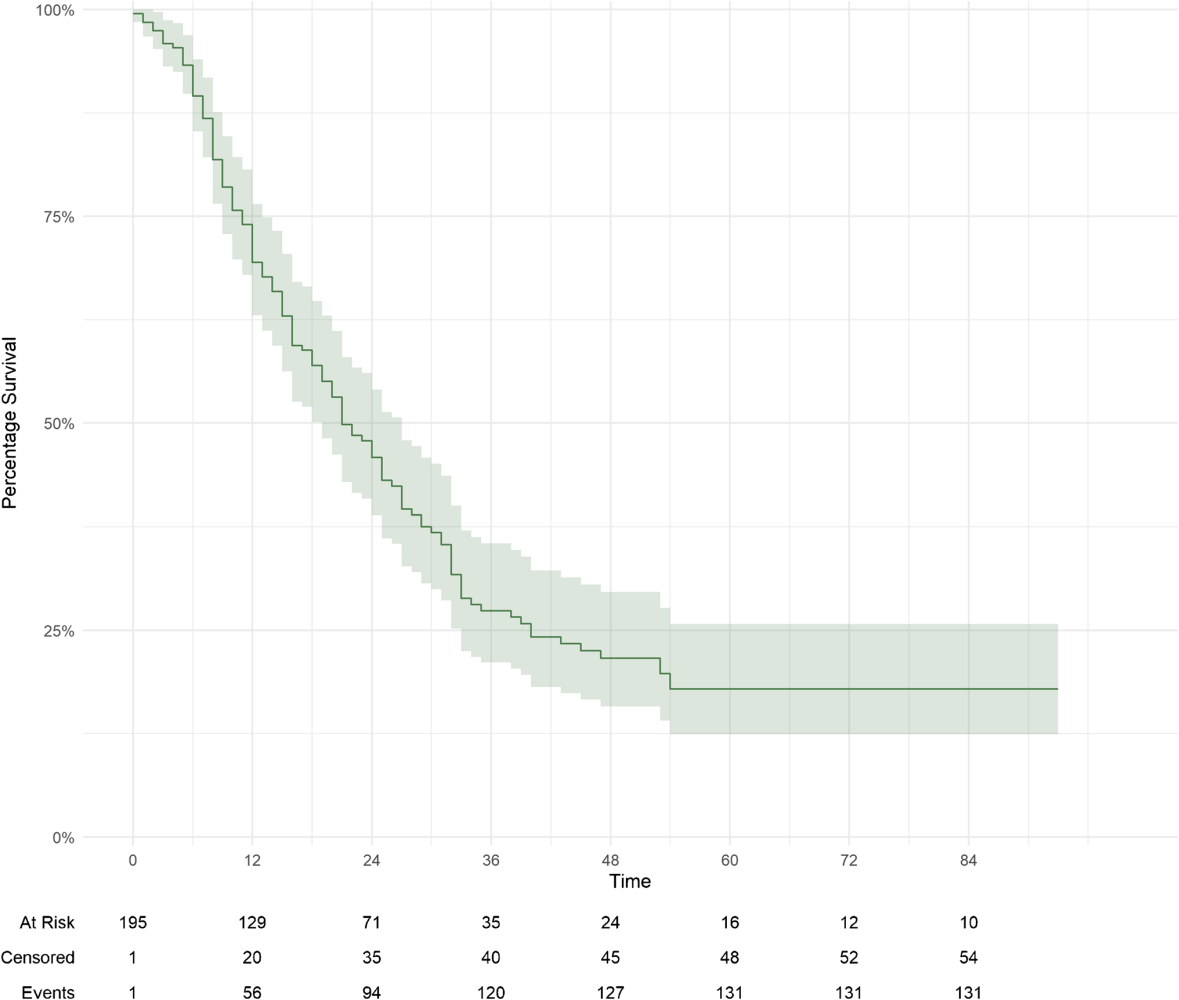
Kaplan-Meier curve demonstrating patient overall survival

**Fig. 3:**
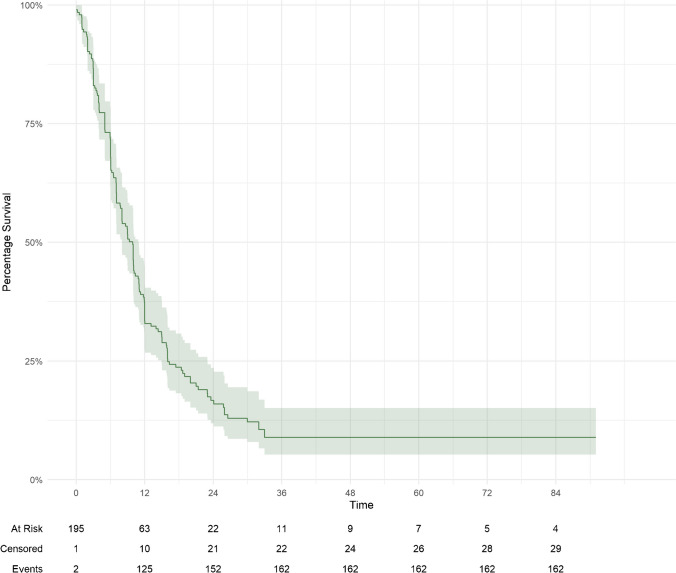
Patient progression free survival via the Kaplan-Meier curve

At the last clinical follow-up, 131 (67.2%) patients were dead, and 64 (32.8%) patients were alive. The median overall survival (OS) from diagnosis of primary breast cancer was 66.3 (40.5-103.8) months and from diagnosis of brain dissemination was 22.0 (12.5–38.3) months. The OS rate at six-, 12-, 24-, 36-, 48-, and 60-months was 90% (95%CI = 85-94%), 69% (95%CI = 63-76%), 46% (95%CI = 39-54%), 27% (95%CI = 21-35%), 22% (95%CI = 16-30%), and 18% (95%CI = 12-26%), respectively (Fig. 2). The PFS rate at six-, 12-, 24-, 36-, 48-, and 60-months was 69% (95%CI = 63-76%), 33% (95%CI = 27-41%), 17% (95%CI = 12-23%), and 8.9% (95%CI = 5.3-15%), respectively (Fig. 3). On multivariate analysis, pertuzumab treatment at the time of SRS was associated with improved OS (HR = 0.50; 95%CI = 0.27–0.94; *p* = 0.032). In this subgroup of patients, GPA scores ≥ 2.5 were associated with increased OS (Suppl. Fig. 1) and primary tumor histology other than invasive ductal and lobular carcinoma was associated with decreased OS (HR = 1.93; 95%CI = 1.17–3.19; *p* = 0.01). OS in patients treated with concurrent trastuzumab was positively correlated with GPA scores ≥ 3.5 and negatively correlated with primary breast cancer histological types other than ductal and lobular carcinomas (HR = 1.89; 95%CI = 1.15–3.13; *p* = 0.012) (suppl. Tables 3 and 4).

### Intra-cranial progression and leptomeningeal dissemination

During follow-up, intracranial progression and leptomeningeal dissemination occurred in 117 (60.0%) and 32 (16.6%) patients, respectively. The median time to leptomeningeal spread was 12.2 (IQR 5.1–22.1) months. In multivariable analysis only age at the time of SRS was negatively correlated with leptomeningeal dissemination (HR = 0.96; 95% CI = 0.93–0.99). In the current study, there was no association between HER-2 targeted therapy and leptomeningeal disease. (Suppl. Table 5)

### Additional management after initial SRS

Post-SRS, 88 (45.8%) patients required additional radiotherapy. due to local SRS failure (*n* = 10) or distant progression (*n* = 78). Of these, 59 patients were treated with SRS, 27 with WBRT and three patients with SRS and WBRT. The median time to first additional radiotherapy was 9.4 months (IQR 4.5–12.9). At a median time of 15.9 months (IQR 5.8–21.8) from SRS twenty-four (12.4%) patients required surgical intervention for tumor progression or ARE. (*n* = 8). (Table 2).

### Adverse radiation events (ARE)

Asymptomatic and symptomatic adverse radiation events (ARE) occurred in 27 (14.0%) and five (2.6%) patients respectively. Symptomatic ARE were classified as mild (Grade 1–2) in three patients and severe in two patients (Grade ≥ 3). The median time to ARE was 6 months (IQR 3–30). Seven patients were treated with a short-course of corticosteroids alone, eight required surgical resection, and two were treated with steroids and bevacizumab. In multivariable analysis, invasive lobular breast carcinoma primary (HR = 3.57, 95% CI = 1.05–12.2) and pertuzumab treatment concurrent with SRS (HR = 4.16, 95% CI = 1.88–9.19) were both significant factors associated with increased risk for overall ARE (Suppl. Table  6) but not for symptomatic ARE.

## Discussion

Many attempts have been made to risk stratify patients with brain metastases according to overall survival, and both breast cancer primary disease and HER2-positive tumor status have consistently emerged as positive prognostic factors [14–19]. One single-institutional experience also reported improved local progression-free survival in patients with HER2-positive cancer, compared with HER2-negative primary tumors [20]. The benefits of monoclonal antibodies such as trastuzumab and pertuzumab are limited in the setting of intracranial disease because of low penetrance of the blood brain barrier [21]. Even so, there is evidence that local therapy, such as stereotactic radiosurgery (SRS), can cause a disruption of the blood brain barrier and allow for increased penetrance of monoclonal antibodies into the brain [22, 23]. 

Small molecule tyrosine kinase inhibitors, including lapatinib, carry the potential to improve outcomes of breast cancer patients with brain metastases, due to the increased penetrance across the blood brain barrier. Concurrent lapatinib with SRS has demonstrated an improved complete response rate in a small study of 84 patients, without evidence for increased toxicity [3]. A phase III trial has shown improved time to progression with the addition of lapatinib in the setting of locally advanced or metastatic HER2-positive breast cancer, and a phase 2 trial showed a partial response rate of 65.9% for brain metastases with the combination of lapatinib and capecitabine [24, 25]. In order to optimize this partial response, however, the combination of SRS and HER2-directed therapy requires further study [26].

This multi-center, retrospective study evaluated the safety and efficacy of SRS in a cohort of 195 HER-2 positive breast cancer female patients treated with SRS for 1706 intracranial metastases. This is the first study to specifically evaluate the effect of SRS on HER-2 breast cancer patients with brain metastases. The current study demonstrates that SRS can afford local tumor control and favorable survival in HER-2 breast cancer patients with brain metastasis.

### Patient overall survival and SRS toxicity

At last follow-up 64 patients were alive. The median overall survival from breast cancer diagnosis was 66.3 months and from brain metastases diagnosis it was 22 months. Our results are in accordance to the median OS reported in previous studies [27–29]. We found pertuzumab treatment at SRS to be associated with increased OS. In patients receiving pertuzumab or trastuzumab at SRS, higher GPA scores were associated with improved OS and primary tumor histology other than invasive ductal and invasive lobular carcinoma was associated with decreased OS.

ARE including radionecrosis are the most serious adverse event of SRS for brain metastases. In line with previous studies, symptomatic adverse radiation events occurred in 2.6% of the patients [3, 29, 30]. The current study suggests that concurrent use of pertuzumab (*p* < 0.001), and invasive lobular carcinoma primary (*p* = 0.042) increased the risk of overall but not of symptomatic adverse radiation effects. The combination of trastuzumab and emtasine treatment has been reported to increase the risk of post-SRS radiation necrosis [6, 31], possibly due to upregulation of Aquaporin-4 channels [31]. However, in our study (*p* = 0.25), and in the study by Mills et al [32], trastuzumab-emtasine administration did not increase the risk of post-SRS radiation necrosis. Improved local tumor control has been noted when combining SRS with immunotherapy or targeted therapy. However, particularly when higher margin doses are prescribed, a higher incidence of radiation necrosis (RN) up to 20%, has also been reported [33]. In the current study, median prescription dose of 16 Gy for small brain metastases with median volume of 0.1cm^3^ was associated with high local tumor control rates and low risk of symptomatic adverse radiation events. Our findings suggest that combining low dose SRS with modern systemic treatments could maintain high tumor control and reduced toxicity compared to the more standard ASTRO doses of 20–24 Gy [34]. The value of reduced dose SRS in long term tumor control needs to be further evaluated in well-designed studies.

### Local and intracranial tumor control

At a median radiological follow up of 14.5 months, local progression occurred in 5.1% of the SRS-treated metastases in our series, similarly to previously reported local failure rates [30, 35]. The local tumor control rate at six-, 12-, 24-, 36- and 60-months was 98%, 94%, 93%, 90%, and 88%, respectively. Factors reported to be associated with improved local control of SRS-treated brain metastases from HER-2 positive breast cancer include lapatinib concurrent with SRS [3, 28, 29], smaller lesion volume [28, 29], and higher prescription dose [28]. In our study pertuzumab treatment at SRS, but not lapatinib, was associated with improved local tumor control of brain metastases. Studies reporting improved local control with SRS concurrent with lapatinib defined concurrent as lapatinib administration on the date of SRS or within five biological half-lives from the date of SRS [3, 29]. In contrast, in our study concurrent therapy was defined as medication administration within four weeks from SRS possibly leading to underestimation of the synergistic effect of lapatinib with SRS.

Despite the high incidence of brain metastases in patients with HER-2 positive breast cancer, current guidelines do not recommend screening neuro-imaging. In our study, as in previous reports [28, 29], tumor volume at SRS was a predictor of local tumor control, further supporting a potential benefit of MRI screening for early detection of brain metastases and subsequent early intervention. Well-designed, randomized studies are required prior to recommending brain MRI screening in this patient population.

Though SRS of brain metastases achieves high local tumor control, the six- and 12-months distant intracranial progression rate is high [3, 7, 9, 30]. Overall intracranial progression occurred in 60% and leptomeningeal disease in 16.6% of the patients in our study. The median progression free survival was 8.9 months. After SRS, 45.8% of the patients required additional radiotherapy, and 12.4% of the patients underwent resection of a brain metatasis. Newer HER-2 targeted therapies have demonstrated increased CNS activity. Trastuzumab-deruxtecan, and tucatinib in combination with trastuzumab and capecitabine, decreased the risk of intracranial progression in the DESTINY-BREAST01 [36] and HER2CLIMB studies, respectively [37, 38]. Our study did not include patients managed with these systemic therapies. The possible synergistic effect of SRS with HER2-targeted therapies with intracranial activity and the feasibility of selective lesion SRS [39] in patients with high total tumor volume needs to be examined in future studies.

### Limitations

Our study is limited by its retrospective nature and patient selection bias at each participating institution. Breast cancer hormone receptor status in HER-2 positive breast cancer is a known factor affecting patient prognosis [40]. Data on hormone receptor status was not available, therefore patients with luminal HER2-positive and HER2-enriched cancers were analyzed together adding confounding biases to the study. In addition, the effect of newer systemic therapies with known CNS activity and their effect in local and intracranial tumor control needs to be evaluated in future studies. Moreover, data on mortality due to intra-cranial and extra-cranial progression were not captured in the study.

## Conclusion

SRS is an effective, low-risk treatment option for selected patients with small volume brain metastases from HER-2 positive breast cancer. Concurrent pertuzumab improved local tumor control and overall survival. In this study, concurrent pertuzumab and SRS appeared to increase the risk for overall but not of symptomatic ARE. Given the survival and local tumor control benefits with an absence of symptomatic ARE, SRS combined with pertuzumab in HER 2 positive breast cancer brain metastasis patients seems to offer a favorable approach.

## Supplementary information

Below is the link to the electronic supplementary material.

ESM 1 (PNG 152 KB)

High Resolution Image (TIF 103 KB)

ESM 2 (DOCX 14.4 KB)

ESM 3 (DOCX 16.4 KB)

ESM 4 (DOCX 19.8 KB)

ESM 5 (DOCX 14.7 KB)

ESM 6 (DOCX 19.3 KB)

ESM 7 (DOCX 18.6 KB)

## Data Availability

The datasets generated during and/or analyzed during the current study are available from the corresponding author on reasonable request.
