# Supplementary figures and images for: Stereotactic radiosurgery for brain metastases from human epidermal receptor 2 positive breast Cancer: an international, multi-center study
Source: J Neurooncol. 2024 Aug 27;170(1):199–208. doi: 10.1007/s11060-024-04775-3 (PMC11446965; doi:10.1007/s11060-024-04775-3)

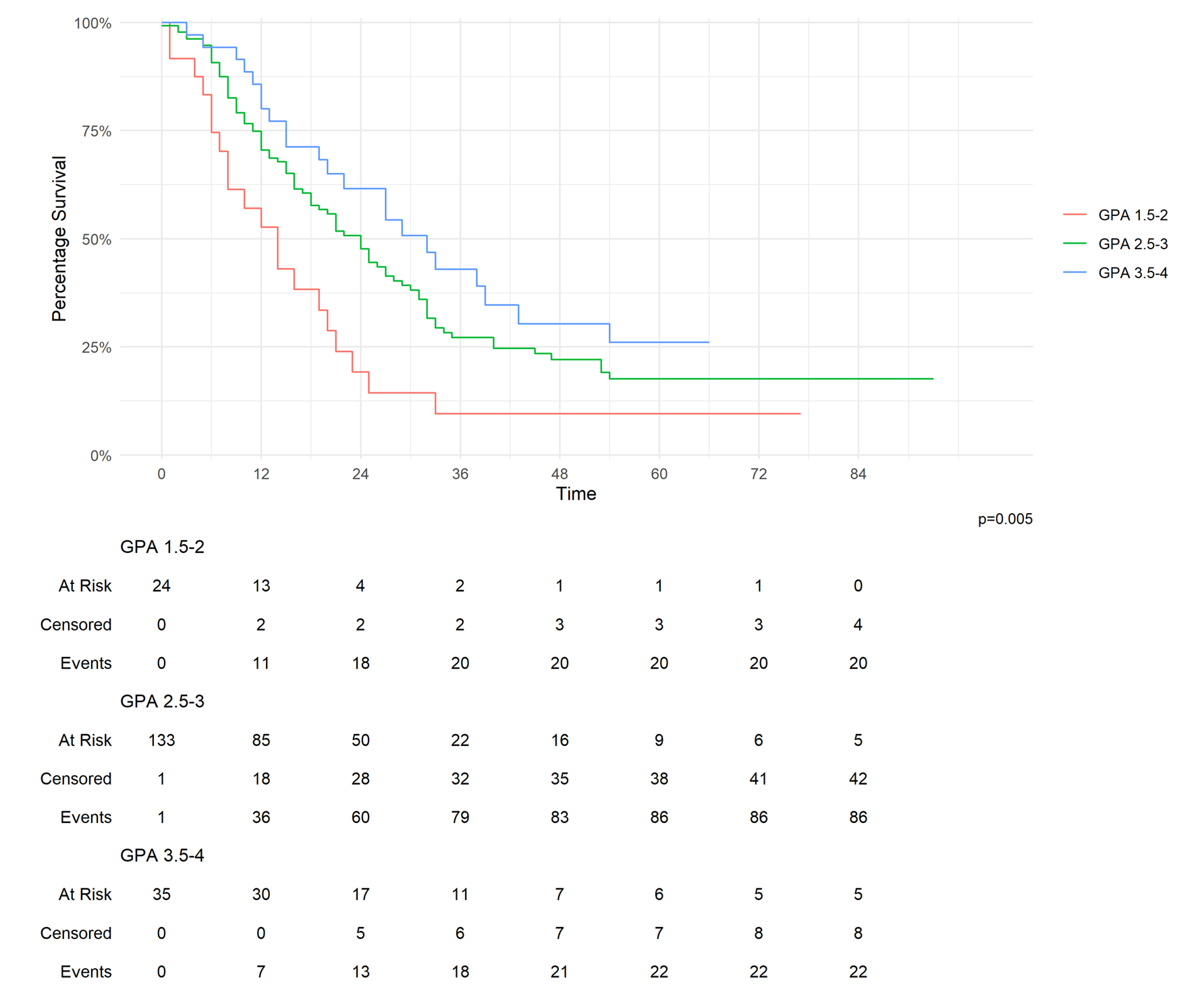

Supplement: Supplementary file 1 — (PNG 152 KB) [file 11060_2024_4775_Fig4_ESM.png]

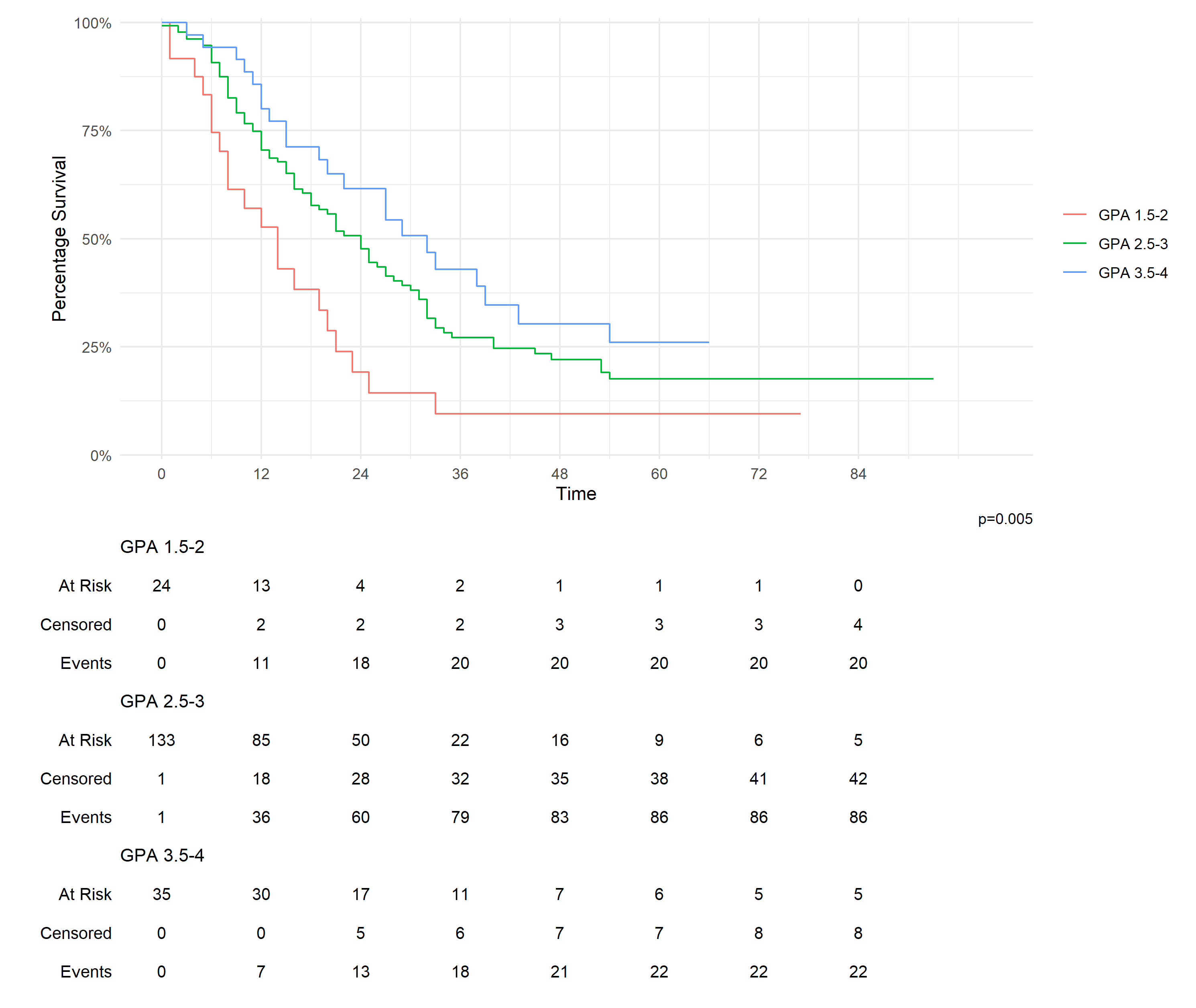

Supplement: Supplementary file 2 — (TIF 103 KB) [file 11060_2024_4775_MOESM1_ESM.tiff]
