# Supplementary material for: Stereotactic radiosurgery for brain metastases from human epidermal receptor 2 positive breast Cancer: an international, multi-center study
Source: J Neurooncol. 2024 Aug 27;170(1):199–208. doi: 10.1007/s11060-024-04775-3 (PMC11446965; doi:10.1007/s11060-024-04775-3)
Supplement: Supplementary file 3 — (DOCX 14.4 KB) [file 11060_2024_4775_MOESM2_ESM.docx]

Supplementary table 1: SRS treatment parameters

| **Median tumor volume, cm** | 0.1 (0.1,0.5) |
| --- | --- |
| Unknown | 35 |
| **Median prescription dose, Gy** | 16.0 (16.0,18.0) |
| Unknown | 1 |
| **Median maximum dose, Gy** | 22.1 (19.0,28.6) |
| Unknown | 2 |
| **Median isodose line, %** | 80.0 (60.0,85.0) |
| Unknown | 1 |
| **Fractions** |  |
| 1 | 1,695 (99.4%) |
| 3 | 5 (0.3%) |
| 5 | 6 (0.4%) |
| **V12** | 0.7 (0.4,2.4) |
| Unknown | 82 |
| *^1^*n (%); Median (25%,75%) | |
