# Supplementary material for: Stereotactic radiosurgery for brain metastases from human epidermal receptor 2 positive breast Cancer: an international, multi-center study
Source: J Neurooncol. 2024 Aug 27;170(1):199–208. doi: 10.1007/s11060-024-04775-3 (PMC11446965; doi:10.1007/s11060-024-04775-3)
Supplement: Supplementary file 4 — (DOCX 16.4 KB) [file 11060_2024_4775_MOESM3_ESM.docx]

Supplementary Table 2: Univariate and multivariate analysis of factors associated with local control.

|  | **Univariable** | | | | **Multivariable** | | |
| --- | --- | --- | --- | --- | --- | --- | --- |
| **Characteristic** | **N** | **HR***^1^* | **95% CI***^1^* | **p-value** | **HR***^1^* | **95% CI***^1^* | **p-value** |
| **Tumor volume** | 1,671 | 1.11 | 1.07, 1.15 | <0.001 | 1.10 | 1.07, 1.14 | <0.001 |
| **Prescription dose** | 1,705 | 0.98 | 0.89, 1.09 | 0.76 | 0.96 | 0.88, 1.06 | 0.46 |
| **Concurrent trastuzumab** | 1,706 |  |  |  |  |  |  |
| No |  | — | — |  |  |  |  |
| Yes |  | 0.79 | 0.51, 1.24 | 0.31 |  |  |  |
| **Concurrent pertuzumab** | 1,706 |  |  |  |  |  |  |
| No |  | — | — |  | — | — |  |
| Yes |  | 0.39 | 0.18, 0.84 | 0.016 | 0.42 | 0.19, 0.92 | 0.029 |
| **Concurrent lapatinib** | 1,706 |  |  |  |  |  |  |
| No |  | — | — |  |  |  |  |
| Yes |  | 0.95 | 0.46, 1.96 | 0.88 |  |  |  |
| **Concurrent emtasine trastuzumab** | 1,705 |  |  |  |  |  |  |
| No |  | — | — |  |  |  |  |
| Yes |  | 0.00 | 0.00, Inf | >0.99 |  |  |  |
| **Concurrented targeted therapy** | 1,706 |  |  |  |  |  |  |
| No |  | — | — |  |  |  |  |
| Yes |  | 0.64 | 0.42, 0.98 | 0.041 |  |  |  |
| *^1^*HR = Hazard Ratio, CI = Confidence Interval | | | | | | | |
