# Supplementary material for: Stereotactic radiosurgery for brain metastases from human epidermal receptor 2 positive breast Cancer: an international, multi-center study
Source: J Neurooncol. 2024 Aug 27;170(1):199–208. doi: 10.1007/s11060-024-04775-3 (PMC11446965; doi:10.1007/s11060-024-04775-3)
Supplement: Supplementary file 6 — (DOCX 14.7 KB) [file 11060_2024_4775_MOESM5_ESM.docx]

Supplementary table 4: Median overall survival rate and median overall survival rates according to GPA at SRS

|  | **6 Months** | **12 Months** | **18 Months** | **24 Months** | **36 Months** | **48 Months** | **60 Months** |
| --- | --- | --- | --- | --- | --- | --- | --- |
| **Overall survival** | 90% (85%, 94%) | 69% (63%, 76%) | 57% (50%, 65%) | 46% (39%, 54%) | 27% (21%, 35%) | 22% (16%, 30%) | 18% (12%, 26%) |
| **Overall survival by GPA** |  |  |  |  |  |  |  |
| GPA 1.5-2 | 63% (46%, 85%) | 21% (9.6%, 45%) | 17% (6.8%, 41%) | 8.3% (2.2%, 31%) | 4.2% (0.6%, 28%) | — (—, —) | — (—, —) |
| GPA 2.5-3 | 65% (57%, 74%) | 28% (21%, 38%) | 20% (14%, 29%) | 16% (10%, 25%) | 9.1% (4.9%, 17%) | 9.1% (4.9%, 17%) | 9.1% (4.9%, 17%) |
| GPA 3.5-4 | 89% (79%, 100%) | 60% (46%, 79%) | 42% (28%, 62%) | 27% (15%, 49%) | 12% (3.9%, 38%) | 12% (3.9%, 38%) | 12% (3.9%, 38%) |
