# Supplementary material for: Stereotactic radiosurgery for brain metastases from human epidermal receptor 2 positive breast Cancer: an international, multi-center study
Source: J Neurooncol. 2024 Aug 27;170(1):199–208. doi: 10.1007/s11060-024-04775-3 (PMC11446965; doi:10.1007/s11060-024-04775-3)
Supplement: Supplementary file 7 — (DOCX 19.3 KB) [file 11060_2024_4775_MOESM6_ESM.docx]

Supplementary table 5: Univariate and multivariate analysis for leptomeningeal progression.

|  | **Univariable** | | | | **Multivariable trastuzumab** | | | **Multivariable pertuzumab** | | |
| --- | --- | --- | --- | --- | --- | --- | --- | --- | --- | --- |
| **Characteristic** | **N** | **HR***^1^* | **95% CI***^1^* | **p-value** | **HR***^1^* | **95% CI***^1^* | **p-value** | **HR***^1^* | **95% CI***^1^* | **p-value** |
| **Histology** | 184 |  |  |  |  |  |  |  |  |  |
| Invasive ductal carcinoma |  | — | — |  | — | — |  | — | — |  |
| Invasive lobular carcinoma |  | 1.17 | 0.51, 2.67 | 0.71 | 0.77 | 0.32, 1.85 | 0.56 | 0.86 | 0.36, 2.05 | 0.73 |
| Other |  | 1.90 | 1.15, 3.13 | 0.012 | 1.89 | 1.15, 3.13 | 0.012 | 1.93 | 1.17, 3.19 | 0.010 |
| **Systemic disease status** | 195 |  |  |  |  |  |  |  |  |  |
| Extracranial metastases |  | — | — |  |  |  |  |  |  |  |
| No extracranial metastases |  | 0.90 | 0.59, 1.37 | 0.62 |  |  |  |  |  |  |
| **KPS at SRS** | 192 |  |  |  |  |  |  |  |  |  |
| 60 |  | — | — |  |  |  |  |  |  |  |
| 70 |  | 0.84 | 0.29, 2.41 | 0.74 |  |  |  |  |  |  |
| 80 |  | 0.68 | 0.24, 1.88 | 0.45 |  |  |  |  |  |  |
| 90 |  | 0.46 | 0.16, 1.30 | 0.14 |  |  |  |  |  |  |
| 100 |  | 0.64 | 0.21, 1.89 | 0.41 |  |  |  |  |  |  |
| **Age at SRS** | 195 | 1.02 | 1.00, 1.03 | 0.030 |  |  |  |  |  |  |
| **GPA score** | 192 |  |  |  |  |  |  |  |  |  |
| GPA 1.5-2 |  | — | — |  | — | — |  | — | — |  |
| GPA 2.5-3 |  | 0.53 | 0.32, 0.86 | 0.010 | 0.53 | 0.31, 0.90 | 0.020 | 0.57 | 0.34, 0.97 | 0.038 |
| GPA 3.5-4 |  | 0.38 | 0.21, 0.70 | 0.002 | 0.38 | 0.20, 0.75 | 0.005 | 0.37 | 0.19, 0.72 | 0.003 |
| **Concurrent trastuzumab** | 195 |  |  |  |  |  |  |  |  |  |
| No |  | — | — |  | — | — |  |  |  |  |
| Yes |  | 0.62 | 0.42, 0.92 | 0.018 | 0.68 | 0.45, 1.04 | 0.072 |  |  |  |
| **Concurrent pertuzumab** | 195 |  |  |  |  |  |  |  |  |  |
| No |  | — | — |  |  |  |  | — | — |  |
| Yes |  | 0.48 | 0.26, 0.89 | 0.020 |  |  |  | 0.50 | 0.27, 0.94 | 0.032 |
| **Concurrent lapatinib** | 195 |  |  |  |  |  |  |  |  |  |
| No |  | — | — |  |  |  |  |  |  |  |
| Yes |  | 1.48 | 0.89, 2.47 | 0.13 |  |  |  |  |  |  |
| **Concurrent emtasine trastuzumab** | 194 |  |  |  |  |  |  |  |  |  |
| No |  | — | — |  |  |  |  |  |  |  |
| Yes |  | 1.05 | 0.43, 2.56 | 0.92 |  |  |  |  |  |  |
| **Concurrented targeted therapy** | 195 |  |  |  |  |  |  |  |  |  |
| No |  | — | — |  |  |  |  |  |  |  |
| Yes |  | 0.82 | 0.58, 1.16 | 0.25 |  |  |  |  |  |  |
| *^1^*HR = Hazard Ratio, CI = Confidence Interval | | | | | | | | | | |
