# Supplementary material for: Stereotactic radiosurgery for brain metastases from human epidermal receptor 2 positive breast Cancer: an international, multi-center study
Source: J Neurooncol. 2024 Aug 27;170(1):199–208. doi: 10.1007/s11060-024-04775-3 (PMC11446965; doi:10.1007/s11060-024-04775-3)
Supplement: Supplementary file 8 — (DOCX 18.6 KB) [file 11060_2024_4775_MOESM7_ESM.docx]

Supplementary table 6: Analysis of factors associated with adverse radiation events.

|  | **Univariable** | | | | **Multivariable all targeted** | | | **Multivariable pertuzumab** | | |
| --- | --- | --- | --- | --- | --- | --- | --- | --- | --- | --- |
| **Characteristic** | **N** | **HR***^1^* | **95% CI***^1^* | **p-value** | **HR***^1^* | **95% CI***^1^* | **p-value** | **HR***^1^* | **95% CI***^1^* | **p-value** |
| **Histology** | 184 |  |  |  |  |  |  |  |  |  |
| Invasive ductal carcinoma |  | — | — |  | — | — |  | — | — |  |
| Invasive lobular carcinoma |  | 2.81 | 0.83, 9.47 | 0.10 | 3.53 | 1.04, 12.0 | 0.043 | 3.57 | 1.05, 12.2 | 0.042 |
| Other |  | 1.92 | 0.72, 5.11 | 0.19 | 2.15 | 0.81, 5.75 | 0.13 | 2.30 | 0.85, 6.21 | 0.10 |
| **Age at SRS** | 195 | 1.00 | 0.98, 1.03 | 0.75 |  |  |  |  |  |  |
| **Total V12Gy** | 195 | 0.98 | 0.95, 1.01 | 0.19 | 0.98 | 0.95, 1.01 | 0.18 | 0.98 | 0.95, 1.01 | 0.17 |
| **GPA score** | 192 |  |  |  |  |  |  |  |  |  |
| GPA 1.5-2 |  | — | — |  |  |  |  |  |  |  |
| GPA 2.5-3 |  | 1.74 | 0.41, 7.41 | 0.45 |  |  |  |  |  |  |
| GPA 3.5-4 |  | 1.65 | 0.34, 7.89 | 0.53 |  |  |  |  |  |  |
| **Concurrent trastuzumab** | 195 |  |  |  |  |  |  |  |  |  |
| No |  | — | — |  |  |  |  |  |  |  |
| Yes |  | 1.41 | 0.69, 2.90 | 0.35 |  |  |  |  |  |  |
| **Concurrent pertuzumab** | 195 |  |  |  |  |  |  |  |  |  |
| No |  | — | — |  |  |  |  | — | — |  |
| Yes |  | 3.64 | 1.69, 7.84 | <0.001 |  |  |  | 4.16 | 1.88, 9.19 | <0.001 |
| **Concurrent lapatinib** | 195 |  |  |  |  |  |  |  |  |  |
| No |  | — | — |  |  |  |  |  |  |  |
| Yes |  | 1.78 | 0.61, 5.19 | 0.29 |  |  |  |  |  |  |
| **Concurrent emtasine trastuzumab** | 194 |  |  |  |  |  |  |  |  |  |
| No |  | — | — |  |  |  |  |  |  |  |
| Yes |  | 2.35 | 0.56, 9.93 | 0.25 |  |  |  |  |  |  |
| **Concurrented targeted therapy** | 195 |  |  |  |  |  |  |  |  |  |
| No |  | — | — |  | — | — |  |  |  |  |
| Yes |  | 2.37 | 1.17, 4.82 | 0.017 | 2.38 | 1.12, 5.04 | 0.024 |  |  |  |
| *^1^*HR = Hazard Ratio, CI = Confidence Interval | | | | | | | | | | |
